# Unravelling the Significance of Extracellular Vesicle‐Associated DNA in Cancer Biology and Its Potential Clinical Applications

**DOI:** 10.1002/jev2.70047

**Published:** 2025-03-16

**Authors:** Jamal Ghanam, Kristína Lichá, Venkatesh Kumar Chetty, Ommolbanin Asad Pour, Dirk Reinhardt, Barbora Tamášová, Peter Hoyer, Jan Lötvall, Basant Kumar Thakur

**Affiliations:** ^1^ Department of Pediatrics III University Hospital Essen Essen Germany; ^2^ AML‐BFM Study Group; ^3^ Institute of Molecular Biomedicine, Faculty of Medicine Comenius University Bratislava Slovakia; ^4^ Department of Pediatrics II University Hospital Essen, University of Duisburg‐Essen Essen Germany; ^5^ Krefting Research Centre, Institute of Medicine, Sahlgrenska Academy University of Gothenburg Gothenburg Sweden

**Keywords:** biomarker, EV‐DNA, EV‐DNA packaging, EV‐DNA topology, extracellular vesicles

## Abstract

Extracellular vesicles (EVs) play a key role in cell‐to‐cell communication and have drawn significant attention due to their potential clinical applications. However, much remains to be understood about the biology of EV‐associated DNA (EV‐DNA). EV‐DNA is actively released by both normal and malignant cells and consists of diverse fragments with varying structures. Because EV‐DNA spans the entire genome of cells from which it originates, it continues to be attractive as a biomarker for cancer diagnosis and monitoring. Further, EV‐DNA delivery can alter the function of recipient cells by interfering with cytoplasmic DNA sensor pathways. This review explores the biology and significance of EV‐DNA, including its topology and fragmentomics features, modality of association with EVs, packaging mechanisms, and potential functions. It also emphasizes the specificity of vesicular DNA in identifying genetic and epigenetic changes in cancer. Additionally, it delves into the impact of EV‐DNA on cellular behaviour and its potential use as a therapeutic target in cancer. The review discusses new insights into EV‐DNA biology and provides perspectives and alternatives to address the challenges and concerns for future EV‐DNA studies.

## Graphical abstract

An overview of the biological functions, structural characteristics, and clinical applications of extracellular vesicle‐associated DNA (EV‐DNA). As a cancer biomarker, EV‐DNA carries genetic and epigenetic modifications, such as SNPs, methylation patterns, and diverse topologies, which aid in cancer diagnosis and monitoring. EV‐DNA plays a crucial role in immune activation by triggering a DNA‐driven immune response through the cGAS‐STING pathway, contributing to immune regulation. In the future, engineered EVs, such as EV‐liposome hybrids, hold promise for delivering EV‐DNA as a potential cancer therapy, highlighting its prospects in clinical applications.

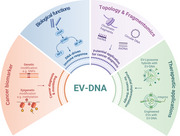

## 1 Introduction

Owing to its unique role as the storage of genetic information, self‐DNA is considered to be restricted to the nucleus and endosymbiont organelles such as mitochondria and chloroplasts. This cell compartmentalization strategy helps maintain and control DNA functions, including replication, gene expression, and DNA damage repair. Already in 1948, Mandel and Métais suggested the existence of cell‐free nucleic acids in human plasma (Mandel and Metais 1948), although it has been unclear for many decades how such DNA is organized in the extracellular space, and which functions it may have. It has become clear that DNA can be passively released from cells during cell death processes such as apoptosis or as a component of extracellular vesicles (EVs) like exosomes released by metabolically active cells (Lo et al. 2021; Schwarzenbach et al. 2011; Thakur et al. 2014; Kahlert et al. 2014; Ghanam et al. 2023). Cells release different types of EVs that carry nucleotides such as RNA or DNA (Valadi et al. 2007; Becker et al. 2016; Ghanam et al. 2022; Lässer et al. 2018). Based on their size and origin in different cellular compartments, EVs can be categorized into two major subtypes: small EVs (e.g., exosomes) or large EVs, also called ectosomes or microvesicles (Welsh et al. 2024). Here, we use the term EVs to describe small vesicles (30–200 nm), which include a heterogeneous population of vesicles such as exosomes derived from the endocytic origin. Evidence has been steadily increasing that cells can actively release DNA in EVs and microvesicles, collectively known as EV‐associated DNA (EV‐DNA) (Ghanam et al. 2022; Malkin and Bratman 2020; Chetty et al. 2022). Recently, chromatin‐like structures, or chromatinized DNA (EV‐chromatin), have been identified as previously undescribed components of EVs (Ghanam et al. 2023 2024).

It has been assumed that most of the EV‐contained DNA in the circulation belong to the category of large EVs, such as apoptotic bodies blebbing from the cell membrane during apoptosis (Grabuschnig et al. 2020; Han and Lo 2021; Yu et al. 2023). However, EV‐DNA can also be released from metabolically active cells, beyond to the cell‐free DNA (cfDNA) released during apoptosis. Circulating cfDNA is being explored as liquid biopsies for cancer diagnosis and monitoring. Recently, EV‐DNA also came into the limelight as a promising biomarker in cancer liquid biopsy, offering a new ray of hope in the field of cancer diagnostics (Ghanam et al. 2022; Malkin and Bratman 2020). Intriguingly, vesicular DNA showed high specificity in detecting genetic and epigenetic alterations in cancer compared to cfDNA, reassuring us about the progress in this crucial area of research (Kim et al. 2022; Zhou et al. 2021; Lee et al. 2018).

Once regarded as a waste disposal pathway, EVs are now considered to be a natural pathway of communication between cells both in homeostasis and in pathological conditions. EVs can migrate from cell to cell and even between organs. These EVs can be taken up by different types of cells, and autocrine or paracrine signalling is induced by the transport of functional proteins, lipids, and nucleic acids into other cells (Becker et al. 2016; Dixson et al. 2023; Chetty et al. 2024). We are now beginning to understand how such cell‐to‐cell EV shuttling of molecules may influence physiology and pathology. In this review, we delve into the intriguing role of EV‐DNA in this communication, sparking curiosity about this lesser‐known aspect of molecular biology. We discuss new insights specifically in the function and importance of EV‐DNA, including how it is associated with EVs, how it may be packaged into EVs, and what potential function it may have in biology. We also highlight the clinical utility of EV‐DNA as a biomarker in cancer.

## 2 EV‐DNA is cfDNA Associated to EVs

cfDNA is DNA found outside the confines of cells, including in interstitial spaces, circulation, or body fluids. This can exist as naked DNA or DNA fragments associated with protein complexes or inside or on the surface of EVs. The release of cfDNA generally falls into two categories: through cell death processes like apoptosis, necrosis, and NETosis, or active release with EVs (Grabuschnig et al. 2020; Han and Lo 2021). This suggests that EV‐DNA constitutes a distinct and likely biologically active fraction of cfDNA. The association of cfDNA with EVs has produced conflicting results, possibly due to the need for a thorough analysis of the fraction of cfDNA carried by EVs (Kahlert et al. 2014; Fernando et al. 2017; Helmig et al. 2015). These variations could be attributed to the experimental handling of the blood specimens, considering the relatively short half‐life of circulating cfDNA (Han and Lo 2021; Lo et al. 2021). EVs also carry DNA inside and outside their lipid bilayer. cfDNA can be packaged inside or attached to the surface of EVs after release into the extracellular space or circulation. Therefore, the term cfDNA will be used in this review to define the EV‐free DNA fragments found in body fluids or cell culture medium, constituting a distinct (from EV‐DNA) population of extracellular DNA.

## 3 Topology, Fragmentomics, and Localization of EV‐DNA and Floating cfDNA

### 3.1 Floating cfDNA

**FIGURE 1:**
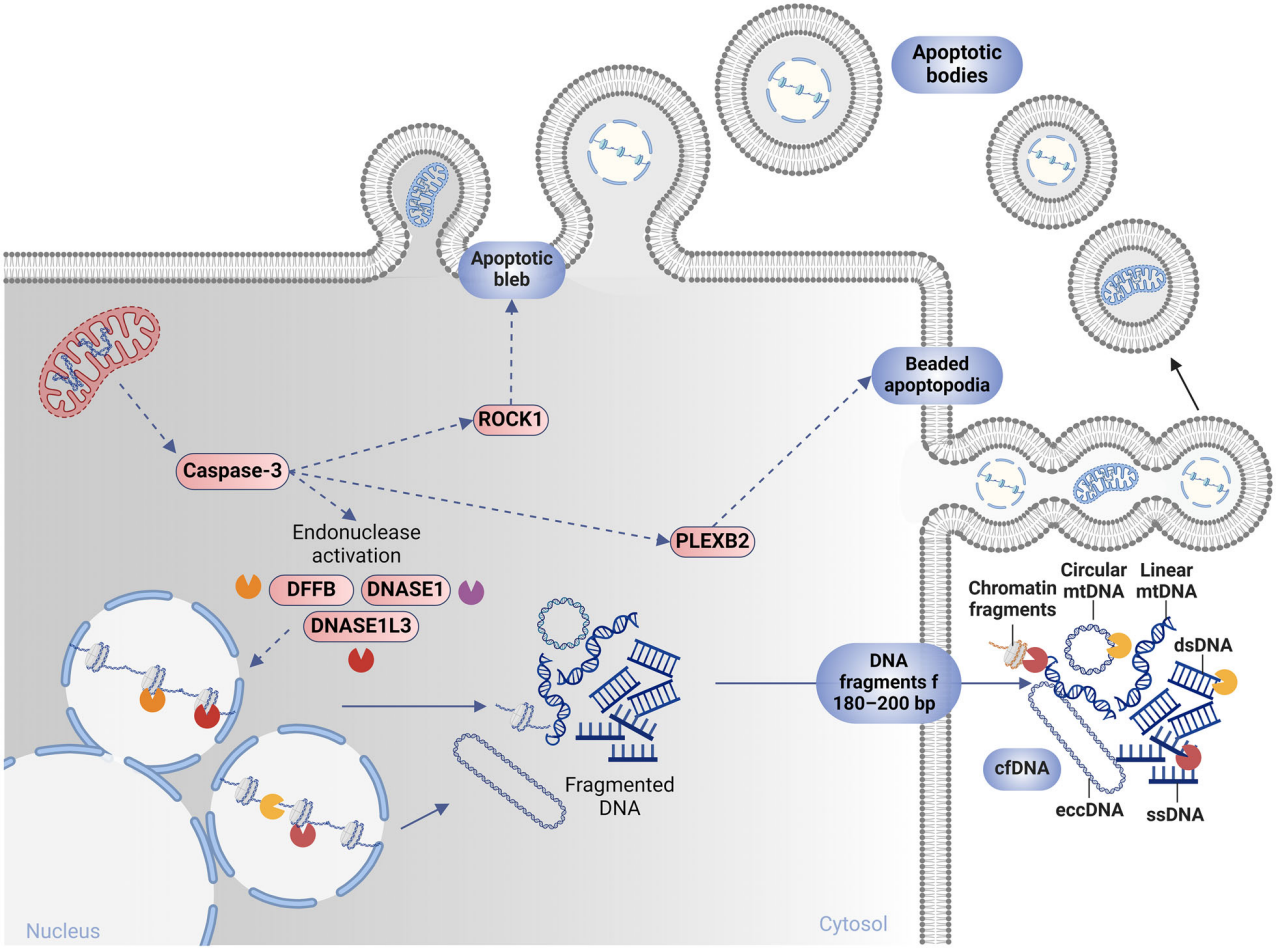
Passive release of cell‐free DNA in apoptotic vesicles. There are multiple modalities for cfDNA release. During apoptosis, cell death leads to the formation of apoptotic bodies that contain nuclear and mitochondrial fragments of DNA inside and/or on the membrane surface. First, Caspase‐3 mediates the cleavage of ROCK1, which induces membrane blebbing and the formation of apoptotic vesicles. Then, the apoptotic membrane protrudes in different types, including the PLEXB2‐mediated beaded apoptopodia that ends in apoptotic vesicle secretion (Atkin‐Smith et al. 2019). Apoptotic cells can also release small DNA fragments (180–200 bp) directly into the surrounding media and circulation. These fragments result from nuclease activation via caspase‐3. Nucleases such as DNA fragmentation factor B (DFFB) and DNASE1L3 are activated within the apoptotic cells, and DNASE1L3 and DNASE1 are also secreted into circulation, where they play a prominent role in the further fragmentation and shaping of plasma cfDNA. Fragmentomics characteristics and end‐motif signatures of cfDNA largely depend on the preferential activity of DFFB, DNASE1L3, and DNASE1 in cutting different sequences.

Floating cfDNA consists of short fragments (40–200 bp) of genomic origin, but mitochondrial DNA (mtDNA) and bacterial DNA sequences may also be detected in blood samples under certain circumstances (Lo et al. 2021; Kustanovich et al. 2019). The characteristics of cfDNA, for example, whether single‐stranded, double‐stranded, or circular, are essential to understanding its function. Further, analysis of DNA topology has revealed the coexistence of linear and circular mtDNA in plasma (Ma et al. 2019). Other types of circular DNA, such as extrachromosomal circular DNA species, have also been identified in human plasma (Figure 1). The circular DNA components have been reported to be longer than the linear molecules and may be more resistant to circulating exonucleases, making it easier to detect. Important aspects of cfDNA topology include its fragmentation patterns, such as fragment sizes (Lo et al. 2010; Underhill et al. 2016), nucleosome architecture (Snyder et al. 2016; Sun et al. 2018), and fragment ends and end motifs (Chan et al. 2016; Sun et al. 2019; Serpas et al. 2019). These studies suggested that the analysis of the fragmentation patterns of cfDNA can help identify its maternal tissues. These patterns are primarily influenced by factors such as nucleosomal organization, chromatin structure, gene expression, and nuclease content of the tissue from which the cfDNA originates. Consistent with this, single‐stranded cfDNA has been found to be more effective than double‐stranded genomic DNA (dsDNA) for detecting short and ultrashort plasma DNA in organ transplant recipients. Unlike dsDNA library preparations, single‐stranded DNA (ssDNA) libraries are more sensitive to ultrashort, degraded cfDNA derived from microbial sources and donor‐derived mtDNA. This enhanced sensitivity can aid in monitoring rejection and infections after transplantation (Vong et al. 2017; Burnham et al. 2016; Moser et al. 2017).

### 3.2 EV‐Associated DNA

**FIGURE 2:**
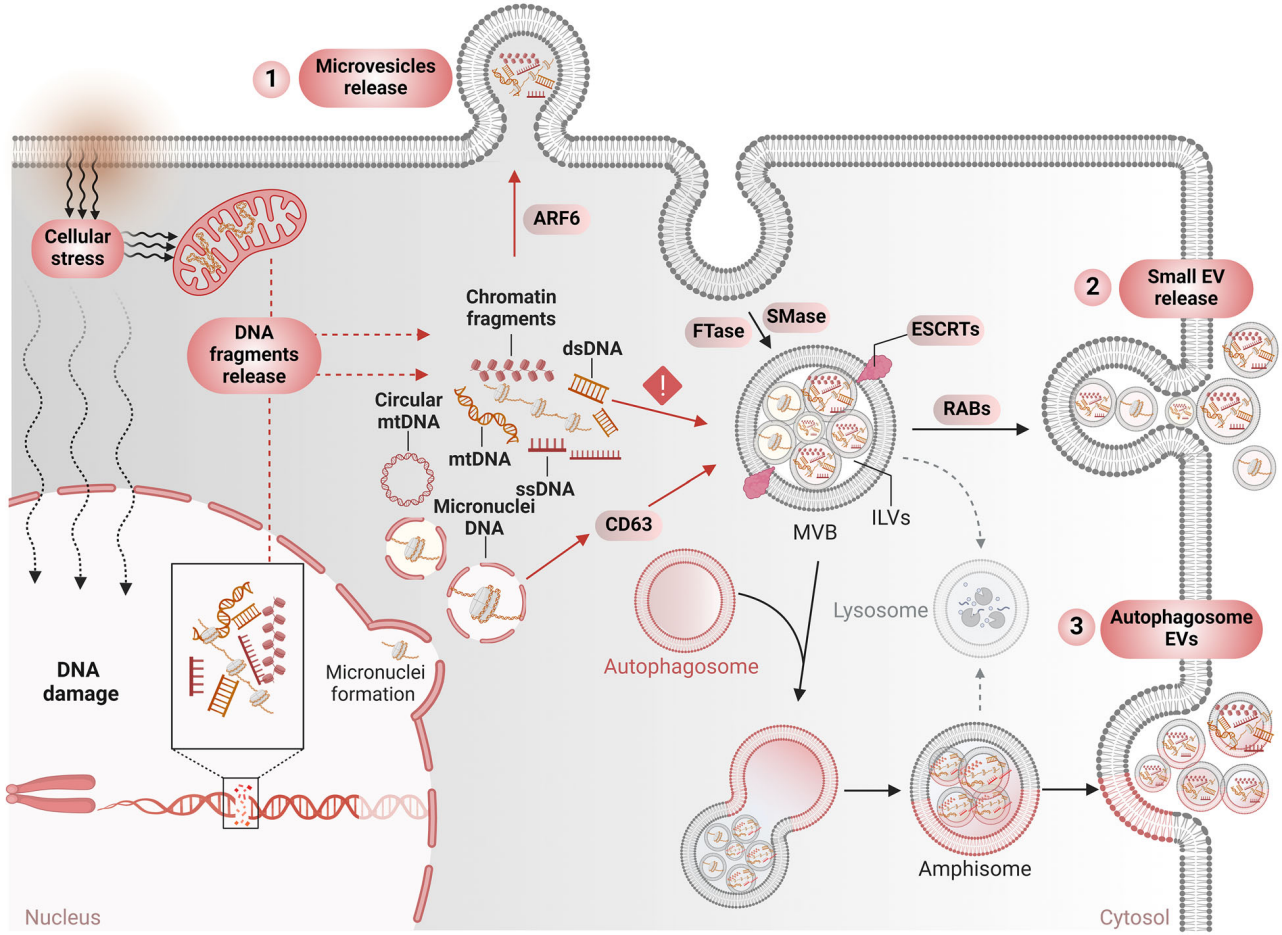
Active release of cfDNA in extracellular vesicles. In response to internal and external stressors, cells release different types of DNA fragments, including micronuclei, to the cytoplasm. Cytoplasmic DNA could be loaded into three different EV populations. (1) The recruitment of cytoplasmic DNA in microvesicles is regulated by ARF6 GTP/GDP cycling and occurs with the cytosolic DNA sensor, cGAS. (2) A variety of cytoplasmic DNA fragments, including ss‐ and dsDNA and chromatinized DNA can be packaged into small EVs that are generated and released via farnesyltransferase (FTase), sphingomyelinase (SMase), and RAB protein pathways. Micronuclei DNA can interact with the EVs marker CD63, which might be a possible mechanism for selecting this DNA for small EVs. So far, ESCRT machinery (endosomal sorting complexes required for transport) responsible for recruiting EV cargo is not involved in EV‐DNA incorporation. (3) Small EVs can also be derived from amphisomes, which are generated from the fusion of autophagosomes and multivesicular bodies (MVB). Amphisome‐derived EVs also contain DNA fragments, but whether they carry the same DNA fragments as the other EV populations needs to be clarified. In general, the modalities of sorting and packaging DNA fragments into small EVs remain largely obscure.

It is known that EVs contain both genomic and mtDNA, but far less is known about the physical characteristics of this DNA, including its fragmentation patterns. EVs carry ssDNA, dsDNA, and mtDNA (Ghanam et al. 2022; Malkin and Bratman 2020; Chetty et al. 2022). While the size of DNA associated with large EVs, such as microvesicles, can reach over 2 million bp, the fragment lengths of EV‐DNA vary from 100 bp to 10 kb (Kahlert et al. 2014; Malkin and Bratman 2020; Vagner et al. 2018; Thakur et al. 2014). Until now, we know that EV‐DNA harbours single‐ and double‐stranded fragments representing the entire genome, including mtDNA (Figure 2) (Kahlert et al. 2014; Chetty et al. 2022; Thakur et al. 2014). Most recently, it has been unambiguously demonstrated that EVs can also transfer chromatinized DNA associated with various proteins, such as histones (Figure 2) (Ghanam et al. 2023).

One of the most important structural features of EV‐DNA is its localization inside or on the surface of EVs, which has been the subject of different results in different works, potentially depending on which cells or systems are studied (Kahlert et al. 2014; Chetty et al. 2022; Thakur et al. 2014; Németh et al. 2017; Jeppesen et al. 2019; Lázaro‐Ibáñez et al. 2019). Many studies have clarified that the surface of the EVs can carry DNA species, but it has also been proposed that DNA can be included in the EV intraluminal space (Kahlert et al. 2014; Ghanam et al. 2023; Chetty et al. 2022; Cai et al. 2013; Lee et al. 2014). Interestingly, the amount of EV‐DNA taken up by recipient cells was not affected by the pre‐treatment of EVs by DNase I or by adding DNase I directly to the culture media to digest cfDNA in real‐time (Ghanam et al. 2023), which suggests that the intraluminal DNA is the component that reaches inside of a recipient cell. However, the question remains how intraluminal and surface DNA are packaged in/on EVs and how they differ in structure and function. To address these questions, more efforts are needed to standardize methods for EV‐DNA characterization, as recently proposed by MISEV2023 guidelines (Welsh et al. 2024) (see more in Box 1). Conversely, the DNA present on the surface may be necessary for the physicochemical properties of the EVs by providing a more pronounced negative charge (Shelke et al. 2016), which may influence how the EVs interact with other EVs or recipient cells. Moreover, there is some evidence suggesting that nucleotides in or on the EVs can activate Toll‐like receptor (TLR), which may provide some activation of innate immunity.

**BOX 1 | EV‐DNA in MISEV2023**

Throughout the last decade, DNA has gained momentum as a novel cargo of many EV populations. Since then, EV‐DNA has demonstrated significant potential as a biomarker, particularly in cancer liquid biopsy (Table 1). Recently, cellular communication via EV‐DNA also came into the limelight as the primary activator of DNA sensing pathways. Different EV isolation approaches can lead to different results when estimating the ratio of DNA enclosed inside or associated with the EV surface. In this regard, MISEV2023 guidelines have already pointed out the need for standardized methods to protect DNA outside the EV during the isolation process (Welsh et al. 2024). The interesting fact is that EV isolation methods can also affect EV‐DNA uptake by recipient cells, as we have previously shown (Chetty et al. 2022). Intriguingly, the amount of EV‐DNA delivered to recipient cells has decreased when polyethylene glycol (PEG) has been used during EV isolation (Chetty et al. 2022). Ultracentrifugation (UC)‐based EV‐isolation methods, combined with precipitation methods such as PEG, may not be ideal for EV‐DNA. However, studies reporting that DNA is mainly associated with EV surface have used UC‐based methods to isolate EVs, as mentioned in the MISEV2023 report (Section 6.5) (Bitto et al. 2017; Liu et al. 2022; Maire et al. 2021; Saari et al. 2020). Considering the whole EV surface cargo, we firmly believe that EV surface DNA is part of the native EV load, which needs to be considered in EV‐DNA studies. In spite of DNA localization, we should instead ask whether surface and intraluminal DNAs have the same topologic characteristics and the same functions in recipient cells. MISEV2023 guidelines have also underlined the necessity of determining the single‐ and double‐stranded (abbreviated as ss‐DNA and dsDNA, respectively) characteristics of any studied EV‐DNA. In this regard, we have previously proposed an enzymatic approach (also summarized in Figure Box 2) to illustrate the presence of ss‐ and dsDNA in EV. Yet, additional exploration of the potential of EV‐DNA for this purpose, including DNA fragmentomics, is warranted, as discussed in the section “Topology, fragmentomics, and localization of cfDNA and EV‐DNA ”.

**TABLE 1:** Detection of cancer related mutations in EV‐DNA and cfDNA.

Source	Isolation method	Disease	Mutation	Genotyping method	Citation
EV‐DNA					
Serum	DNeasy blood and tissue kit (Qiagen)	Pancreatic ductal adenocarcinoma	KRAS TP53	PCR and Sanger sequencing	(Kahlert et al. 2014)
Plasma Pleural fluid	MagAttract HMW DNA Kit (Qiagen)	Pancreaticobiliary cancers	KRAS BRCA2	WGS WES	(San Lucas et al. 2016)
Urine	QIAamp MiniElute kit (Qiagen)	Bladder cancer	KRAS VHL RNF213 and more	PCR and Sanger sequencing	(Zhou et al. 2021)
Blood	DNeasy Blood & Tissue Kit (Qiagen)	Glioma	IDH1^G395A^	Fast cold‐PCR followed by Sanger sequencing	(García‐Romero et al. 2017)
Bronchoalveolar lavage fluid	High‐Pure PCR Template Preparation Kit (Roche Diagnostics)	Non‐small cell lung cancer	EGFR	PNAClampTM EGFR Mutation Detection Kit (Panagene)	(Hur et al. 2019)
Plasma	QIAamp Circulating Nucleic Acid Kit (Qiagen)	Pancreatic ductal adenocarcinoma	KRAS	ddPCR	(Kamyabi et al. 2020)
Serum	QIAamp Circulating Nucleic Acid Kit (Qiagen)	Pancreatic ductal adenocarcinoma	KRAS	ddPCR	(Castillo et al. 2018)
Plasma Serum	QIAamp DNA Micro Kit (Qiagen)	Pancreatic ductal adenocarcinoma	KRAS	ddPCR	(Wang et al. 2020)
Plasma	QIAamp DNA Micro Kit (Qiagen)	Acute myeloid leukaemia	NPM1 FLT3/TKD	GeneScan‐based fragment‐length analysis	(Kontopoulou et al. 2020)
Plasma	Isopropyl alcohol mixture	Renal cell carcinoma	Mitochondrial gene regions HV and CYB	NGS	(Arance et al. 2021)
	**EV‐DNA and cfDNA**				
Plasma	ExoLution Plus Isolation Kit (Exosome Diagnostics) ^a^ ^#^	Multiple advanced cancers	BRAFV^600^ KRAS^G12/G13^ EGFR^exon19del/L858R^	ddPCR	(Möhrmann et al. 2018)
Bronchoalveolar lavage fluid	High‐Pure PCR Template Preparation Kit (Roche Diagnostics)	Non‐small cell lung cancer	EGFR	Peptide nucleic acid‐mediated Real‐Time PCR	(Hur et al. 2019)
Plasma	ExoLution Plus Isolation Kit (Exosome Diagnostics) ^a^ ^#^	Multiple advanced cancers	BRAFV^600^ KRAS^G12/G13^ EGFR^exon19del/L858R^	EV‐DNA: NGS cfDNA: ddPCR and BEAMing PCR	(Möhrmann et al. 2018)
Plasma	EV‐DNA: SeleCTEV Enrichment Kit (Exosomics SpA) cfDNA: QIAamp Circulating Nucleic Acid Kit (Qiagen)	Melanoma	BRAF^V600E^	Digital PCR	(Zocco et al. 2020)
Urine	QIAamp DNA Minikit (Qiagen) ^#^	Urothelial bladder cancer	ARID1A PIK3CA RB1 KMT2D TP53	Shallow WGS	(Lee et al. 2018)
Plasma	EV‐DNA: QIAamp DNA Microkit (Qiagen) cfDNA: QIAamp Circulating Nucleic Acid Kit (Qiagen)	Oropharyngeal squamous cell carcinoma	HPV detection	ddPCR	(Nguyen et al. 2020)
Plasma	EV‐DNA: DNeasy Blood & Tissue Kit (Qiagen) cfDNA: QIAamp Circulating Nucleic Acid Kit (Qiagen)	Advanced pancreatic cancer	KRAS mutation	ddPCR	(Lapin et al. 2023)
Serum (EV‐DNA) Plasma (cfDNA)	MagMAX Cell‐Free DNA Isolation Kit (Thermofisher) ^#^	Colon carcinoma	TP53 p.R175H; APC p.R1450X; TP53 p.R248W; TP53 p.R273H; KRAS p.G12S; PIK3CA p.E545K; PIK3CA p.H1047L; TP53 p.R175H; and more	Targeted NGS	(Thakur et al. 2021)
Plasma	EV‐DNA : QIAmp DNA Micro Kit (Qiagen) cfDNA: QIAamp Circulating Nucleic Acid Kit (Qiagen)	Melanoma	BRAF^V600E^ cKITD816V	ddPCR	(Klump et al. 2018)
Plasma	QIAamp Circulating Nucleic Acid Kit (Qiagen) ^#^	Pancreatic adenocarcinoma	KRAS	ddPCR	(Bernard et al. 2019)
Plasma Tumour tissue ^b^	QIAamp MinElute ccfDNA Mini Kit (Qiagen)	Neuroblastoma	TAF11 ASB11 TMEM14B (EV‐DNA at onset) NPAS4 DTNA NUAK1 (EV‐DNA at relapse) MYCN LCE2D P2RY2 (tumour DNA) and more	WGS	(Degli Esposti et al. 2021)
Pleural effusions	High‐Pure PCR Template Preparation Kit (Roche Diagnostics) ^#^	Pulmonary adenocarcinoma	EGFR	PNAClampTM EGFR Mutation Detection Kit (Panagene)	(Lee et al. 2018)
Blood Plasma **^c^**	QIAamp DNA Mini kit (Qiagen) ^#^	Serous epithelial ovarian cancer	mtDNA copy numbers	Real time quantitative PCR	(Keserű et al. 2019)

^#^ Same isolation method for cfDNA and EV‐DNA.

^a^ Coisolation of DNA and RNA associated with EVs.

^b^ Comparison of EV‐DNA and tumour tissue DNA.

^c^ Comparison of mt‐EV‐DNA, cf‐mt‐DNA, and mt‐DNA.

Abbreviations: ddPCR: droplet digital PCR; HPV: human papilloma virus; NGS: next generation sequencing; WES: whole exome sequencing; WGS: whole genome sequencing.

Unlike cfDNA, the EV lipid bilayer protects EV‐DNA from enzymatic degradation in the circulation, preserving its native physical characteristics. This unique feature of EV‐DNA underscores its potential in clinical applications. Deciphering the topologic and fragmentomics features of EV‐DNA may be key to unlocking new insights. A key consideration is whether DNase treatment of EVs truly removes all surface DNA or whether some is protected by surface protein or an EV protein corona. Regardless, the DNA protected from DNase degradation seems to be the component that can be shuttled into other cells.

In recent years, cfDNA has been explored as a liquid biopsy biomarker, especially in cancer, as it may provide early indications of, for example, cancer relapse and/or metastasis (Schwarzenbach et al. 2011). Importantly, cfDNA can be found free in circulation, bound to proteins like histones, in the form of neutrophil extracellular traps (be part of neutrophil extracellular traps) (Duvvuri and Lood 2019). EVs have also been shown to contain different biomolecules, including DNA. Contrary to cfDNA, which is mostly of nuclear origin, EVs can contain both nuclear and mtDNA inside vesicles or on the outer membrane (Kahlert et al. 2014; Thakur et al. 2014; Lázaro‐Ibáñez et al. 2019; Sansone et al. 2017). Unlike cfDNA, the double membrane of EVs could protect the DNA while in circulation and could be transported into distant cells to maintain homeostasis in health and disease.

**BOX 2 | Experimental strategies to study EV‐DNA localization and topology**

Purified EVs are usually treated with DNase I to study EV‐DNA localization, followed by DNA extraction, quantification, and characterization. However, we have recently utilized a new approach that involves adding the DNase I directly to the media during cell culture, which enables a real‐time degradation of cell‐free DNA that might attach to the surface of EVs. In general, a decrease in DNA concentration upon DNase I treatment indicates the presence of unprotected EV surface DNA. It is worth noting that the DNase I dose (unit) and treatment time should be optimized to digest the DNA carried outside the EVs to small and undetectable nucleotides. In many of the aforementioned studies, EV‐DNA has been quantified using spectrophotometry, which needs to be more specific and sensitive. Therefore, we highly recommend using fluorescence‐based methods that are more sensitive and specific. The use of specific fluorescent dyes enables the quantification of ss‐ and ds‐DNA separately, which reduces false signals generated by contaminants like proteins and RNAs. To investigate the topology of EV‐DNA, DNase I digestion would not be the method of choice as DNase I non‐specifically digests ss‐ and ds‐DNA. Therefore, the current gold standard involves the use of a double‐enzymatic approach, including an S1 nuclease that digests ssDNA and a shrimp dsDNase that specifically hydrolyses dsDNA.

To check for the presence of circular DNA, serial digestion with nucleases and restriction enzymes such as BfaI and MspI can be performed. Digested DNA ends can then be analysed by sequencing to distinguish between linear and circular DNA, as illustrated in Figure Box 6.

## 4 EV‐DNA and cfDNA for Liquid Biopsy: Can They Complement Each Other?

cfDNA can be detected in several body fluids such as blood, plasma, serum, urine, cerebrospinal fluid, or saliva (Schwarzenbach et al. 2011). Changes in the amount of circulating cfDNA are significantly higher in patients with cancer (up to 1000 ng/mL) compared to individuals without cancer (up to 100 ng/mL) (Schwarzenbach et al. 2011; Han and Lo 2021). However, fluctuations in cfDNA content are not only observed in cancer, as high plasma cfDNA levels are detected during pregnancy and after organ transplantation (Lo et al. 2021). The level of cfDNA is also determined by circulating endonuclease activity and the rate of degradation and clearance by the liver and kidney (Schwarzenbach et al. 2011; Rodriguez et al. 1997). Therefore, cfDNA has a highly variable half‐life, ranging from 15 min to multiple hours. The nuclease activity in blood may be a determinant factor for cfDNA turnover, but this aspect of cfDNA physiology remains elusive and needs further investigation. Basically, nothing is known about cfDNA within tissues, especially EV‐associated DNA, and how it may participate in cell‐to‐cell communication.

Accumulating observations suggest that cancer cells secrete more EVs with higher DNA content than non‐cancer cells (Ghanam et al. 2022; Malkin and Bratman 2020). EVs are also more abundant in the plasma of cancer patients, and their number correlates with disease progression in some clinical experiments (Zhou et al. 2021; Yamamoto et al. 2023). As stated earlier, these EVs contain both genomic and mtDNA enclosed inside the vesicles or associated with the outer surface of the lipid bilayer. Unlike cfDNA, EV‐DNA size ranges from 200 to 10 kb, suggesting a non‐apoptotic origin of this DNA. However, estimating the release rate of EVs and their elimination half‐life in blood and other body fluids is complicated. The EV release rate in mice has been estimated to be 18 µg/min, but with a very high turnover (Matsumoto et al. 2020). Thus, the clearance of self‐produced EVs, or intravenously injected EVs, is much slower from the plasma of macrophage‐depleted mice, indicating a balance between high secretion and rapid clearance of blood EVs, most likely in the liver (Imai et al. 2015; Yamamoto et al. 2023; Matsumoto et al. 2017). However, further research is needed to fully understand the mechanisms of macrophage‐mediated clearance of EVs in the liver. The high turnover rate of EVs means that changes in EV populations and their cargo, including DNA fragments, can change within short periods. However, this could offer the possibility to monitor disease progression, including the clonal evolution of tumours, as well as any treatment responses in the future. Regarding biomarkers, cfDNA can be isolated in an easy one‐step method from blood or other biological fluids, whereas specific isolation of EV‐DNA would require a preceding EV isolation step. These purification steps might result in low DNA recovery from EVs, leading to poor sensitivity in detecting certain cancer‐related mutations. However, simplified isolation methods may become available to isolate EVs (free of cfDNA) with high DNA yield, with the first step being size‐exclusion chromatography (Ghanam et al. 2023; Chetty et al. 2022; Zocco et al. 2020).

Numerous groups have examined EV‐DNA and cfDNA to detect genetic anomalies related to cancer. Intriguingly, KRAS mutations have been detected with higher percentages in EV‐DNA, compared to cfDNA, derived from patients with localized, locally advanced, and metastatic pancreatic ductal adenocarcinoma (Allenson et al. 2017). The same trend was observed when evaluating EV and cfDNA from control samples (Allenson et al. 2017). Yang et al. detected KRAS and TP53 mutations in EV‐DNA from the serum of patients with pancreas‐associated pathologies, including pancreatic ductal adenocarcinoma, chronic pancreatitis, and intraductal papillary mucinous neoplasm. Out of 48 serum samples, they detected KRAS mutation in 39.8% and *TP53* mutation in 4.2% (Yang et al. 2017). In the same study, the authors analysed EV‐DNA from 114 healthy subjects and detected 2.6% KRAS mutation, but no TP53 mutation was found (Yang et al. 2017). This finding suggests the potential prognostic value of EV‐DNA (Yang et al. 2017). In a prospective cohort of pancreatic cancer patients, droplet digital PCR has been used to screen for KRAS mutations in cfDNA and EV‐DNA concerning clinical outcomes during the disease progression (Bernard et al. 2019). Unlike cfDNA, high levels of EV‐DNA were reported to be in association with disease progression in patients with potentially resectable tumours following neoadjuvant therapy (Bernard et al. 2019). Moreover, the authors noted a decrease in KRAS mutant allele fraction only specifically in EV‐DNA after surgical resection. This study showed how longitudinal monitoring using liquid biopsy based on EV‐DNA and cfDNA provides valuable predictive and prognostic information relevant to the stratification of therapeutic approaches for pancreatic cancer.

In a study involving a small group of glioma patients, García‐Romero et al. have successfully detected the IDH1^G395A^ mutation in DNA isolated from peripheral blood EVs using ColdPCR (García‐Romero et al. 2017). This mutation was found in 80% of low‐grade gliomas and 40% of high‐grade gliomas (García‐Romero et al. 2017). Interestingly, data from the mutational analysis of EV‐DNA reflected those of tumour immunohistochemistry (IHC, the standard of gold technique in glioma) and conventional PCR performed on solid biopsy samples (García‐Romero et al. 2017). In the same context, DNA from circulating EVs of glioma patients has been compared to histological and radiological characteristics of tumours (Piazza et al. 2022). It has been observed that high EV‐DNA levels were present in the plasma of patients with low‐grade gliomas, and this was positively correlated with tumour volume (Piazza et al. 2022). Conversely, a negative correlation between plasma EV‐DNA and tumour volume was found in the high‐grade glioma group (Piazza et al. 2022). In addition to the mutational status, EV‐DNA has been found to reflect the genome‐wide methylation and copy number variation (CNV) of glioblastoma cells, allowing for their molecular classification (Maire et al. 2021). Currently, no study has compared or combined information from EV‐DNA and cfDNA for the management of gliomas or other CNS pathologies. However, cfDNA has been used in various studies for screening, diagnosis, classification, monitoring, and detecting relapse of CNS cancers, as reviewed by Mair and Mouliere (Mair and Mouliere 2022). Recent genomic analysis of vesicular DNA extracted from in vitro and in vivo models of metastatic prostate cancer (mPC) has shown that EV‐DNA reflects corresponding tissue biopsies and circulating tumour cfDNA and is associated with clinical progression of mPC (Casanova‐Salas et al. 2024).

Small EVs are ubiquitous in human body fluids, and targeted isolation of EVs from specific body fluids seems to be more relevant to monitor certain types of cancer. In this respect, Kim et al. conducted a large‐scale prospective study comparing the effectiveness of tissue‐based genotyping, plasma cfDNA, and EV‐DNA derived from bronchoalveolar lavage fluid (BALF) in monitoring EGFR mutations in advanced non‐small cell lung cancer (NSCLC) patients (Kim et al. 2022). The study found that information obtained from BALF EV‐DNA showed high compliance in terms of sensitivity, specificity, and concordance with standard tissue‐based genotyping for detecting EGFR mutations (Kim et al. 2022). At the same time, cfDNA‐based data demonstrated lower rates of compliance (Kim et al. 2022). The same group had previously indicated that the detection sensitivity of EGFR mutations increases with tumour stages, likely due to increased levels of EV‐DNA released in BALF as the disease progresses (Hur et al. 2019, Kim et al. 2023). In a similar context, EV‐DNA showed high specificity in detecting mutations associated with bladder cancer when derived from urine EVs, compared to plasma EVs or plasma cfDNA (Zhou et al. 2021, Lee et al. 2018). Particularly, whole‐exome sequencing of EV‐DNA from urine and serum EVs, bladder tumours, and peripheral blood mononuclear cells revealed exonic and 3′ UTR variants in frequently mutated genes in bladder cancer, detectable in urine EV‐DNA and matched tumour samples (Zhou et al. 2021). Nonetheless, this study did not include cfDNA as an analyte. In another study, Lee et al. (2018) compared the mutation status of cfDNA and EV‐DNA from the urine of urothelial bladder cancer patients (Lee et al. 2018). As a reference for mutation analysis, they used genomic profiling of tumour samples and matched it with urinary cfDNA and EV‐DNA (Lee et al. 2018). Of the 17 found genetic mutations, 14 were identified in cfDNA and 12 were identified in EV‐DNA using shallow whole genome sequencing. The CNV plots of tumour samples and cfDNA or EV‐DNA showed a similar pattern (Lee et al. 2018).

In addition to genomic DNA, mtDNA is also found as part of EV‐DNA (EV‐mtDNA) and cfDNA, and it is emerging as a potential cancer biomarker. Sansone et al. suggested that the existence of ND1 EV‐mtDNA in circulating EVs is specific to individuals with HTR disease and does not merely represent the extent of metastatic disease (Sansone et al. 2017). Fragments of mitochondrial genes such as HV1 and CYB have been detected in EVs isolated from the plasma of patients with renal cell carcinoma (RCC) (Arance et al. 2021). The analysis of the relationship between these EV‐mtDNA markers and the clinical outcomes showed their relevance for both screening and aggressiveness assessment of RCC (Arance et al. 2021). The copy numbers of mtDNA in patients with serous epithelial ovarian cancer were compared across whole blood, EVs, and cell‐free mtDNA (Keserű et al. 2019). In late‐stage cancer patients, it was found that the copy number of mtDNA in EVs was the highest, followed by plasma mtDNA and whole blood mtDNA. Significant differences were observed between the mtDNA copy numbers of healthy individuals and the EVs or whole blood mtDNA of cancer patients (Keserű et al. 2019). EV‐mtDNA was also investigated for its potential role as a biomarker for glioblastoma (Soltész et al. 2022). The authors have compared mtDNA copy numbers and point mutations in brain tissue DNA and plasma‐derived EV‐DNA of glioblastoma patients and control individuals (Soltész et al. 2022). They revealed differences in copy numbers and point mutations between patients and controls when mtDNA was derived from both tumour tissues and plasma EVs, suggesting the potential use of EV‐mtDNA as a biomarker (Soltész et al. 2022). The use of EV‐DNA as early biomarkers in cancer has garnered attention. However, there are several key challenges that need to be addressed in order to bring EV‐DNA‐based diagnostic tests into routine clinical use. These challenges include finding methods to isolate and extract EV‐associated DNA without compromising its quantity and quality for further genetic and epigenetic analysis. Furthermore, it is important to determine whether specific DNA mutations, epigenetic modifications, or gene expression patterns can be reliably detected and quantified in EV‐associated DNA. Additionally, enhancing the sensitivity and specificity of EV‐associated DNA analysis for biomarker discovery and clinical diagnostics is crucial, as the heterogeneity of the blood material containing DNA from healthy and tumour origin makes it difficult to filter cancer‐specific signals, similar to cfDNA. Standardization and validation of EV‐associated DNA isolation and analysis as a biomarker will ensure reproducibility and comparability across different studies, overcoming the limitations and challenges associated with analysing EV‐associated DNA from different body fluids other than the classical blood plasma or serum materials, such as urine or cerebrospinal fluid. Once EV‐DNA‐based technology is established, it will serve as a valuable tool in the field of cancer by providing information about “real time” disease progression, treatment response, and minimal residual disease.

**FIGURE 3:**
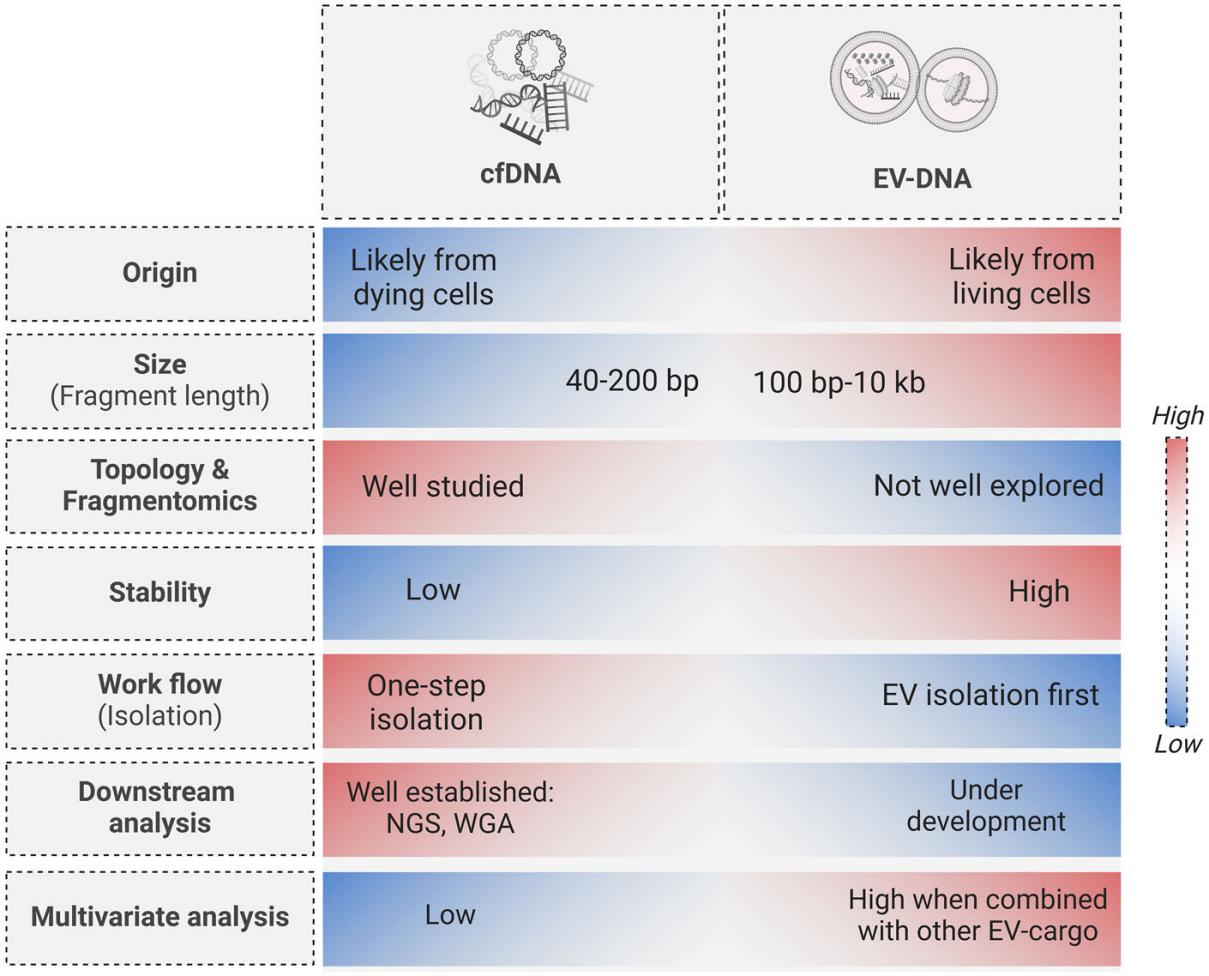
Comparative analysis of EV‐DNA and cfDNA characteristics. In cancer, cfDNA release is largely attributed to cell death processes such as apoptosis and necrosis, though active secretion cannot be excluded (Schwarzenbach et al. 2011; Han and Lo 2021). In contrast, EVs, as defined here, are released by metabolically active cells, with their associated DNA likely originating from living cells. While cfDNA is well‐studied with simpler workflows, it suffers from low stability and limited multivariate analysis. EV‐DNA, on the other hand, offers higher stability, larger fragment sizes, and the potential for multivariate analysis when combined with other EV cargo. However, critical aspects of EV‐DNA research, including fragment patterns and analytical methodologies, remain to be thoroughly explored and standardized. Addressing these gaps is essential for establishing EV‐DNA as a reliable biomarker in cancer diagnostics. Once resolved, EV‐DNA could provide valuable insights, bridging the current knowledge gap in cfDNA research and advancing the utility of liquid biopsy in oncology.

cfDNA appears to be more cost‐effective than EV‐DNA that requires an isolation step of EVs. However, the majority of cfDNA comes from dying cells with topologic and physical modifications due to nuclease degradation in circulation. In contrast, EV‐DNA released from metabolically active cells is protected from nuclease digestion, providing a more accurate reflection of the disease state and highlighting tumour dynamics. Nevertheless, analysing DNA from both the cell‐free and EV compartments will complement each other in obtaining cancer‐specific information from patients (more in Figure 3).

## 5 Biogenesis and Packaging Mechanism of EV‐DNA: What do We Know So Far?

The latest developments in EV‐RNA research have enhanced our understanding of EV‐DNA biology, as outlined in the MISEV2023 guidelines (Welsh et al. 2024). The concentration of DNA and RNA in EVs varies depending on the type and the physiological/pathological state of the cell they originate from, indicating that they are enriched in EVs under particular circumstances (O'Brien et al. 2020; Luo et al. 2021; Takahashi et al. 2017). It is conceivable that EVs contain different types of RNA (mRNA, lncRNA, and miRNA) and DNA (ssDNA, dsDNA, and chromatin) fragments (Ghanam et al. 2023; Thakur et al. 2014; O'Brien et al. 2020). This could imply the existence of specific mechanisms for sorting into RNA and DNA EVs, which are not fully defined, especially in the case of DNA. For example, EVs released from neurons and astrocytes have different miRNA signatures (Luo et al. 2021). Specifically, neuronal EVs contain more miRNAs than astrocytic EVs with specific sequence motifs called EXOmotifs. In a study by Gámbaro et al. (2020), cells were transfected with synthetic stable oligonucleotides, glycine 5ʹ tRNA halves, to track how RNA is loaded into EVs (Gámbaro et al. 2020). The researchers found that the concentrations of tRNA halves in vesicles and cells were proportional to the transfection concentrations, indicating a concentration‐driven loading mechanism (Gámbaro et al. 2020). Additionally, RNA stability (resistance to RNase degradation) appears to be an essential factor in packaging RNA into EVs (Gámbaro et al. 2020). In contrast, it is not known whether a deficiency in nuclease activity (such as TREX1) affects EV‐DNA loading. Current opinions suggest that RNAs are either passively loaded due to high abundance or actively targeted to the endosomal compartment and incorporated into intraluminal vesicles, as extensively discussed by O'Brien et al. (2020). However, the exact cellular mechanisms involved in sorting and loading RNAs into EVs remain elusive.

Similarly, the biology of EV‐DNA and its packaging mechanisms have yet to be explored. For instance, whether DNA is associated with all EV populations still needs to be discovered. Interestingly, Lázaro‐Ibáñez et al. analysed the DNA content of different EV subpopulations and found more DNA in high‐density EVs (Lázaro‐Ibáñez et al. 2019). Before this discovery, it was argued that only a low proportion of EVs carry DNA. Finally, these studies still had some limitations, such as thoroughly characterizing the topology and fragmentomics of EV‐DNA in line with EV populations. Further, questions related to cellular mechanisms of EV‐DNA packaging remain unanswered. Despite that, a few seminal studies have provided some insights.

Yokoi et al. proposed a mechanistic explanation involving micronuclei as a source of EV‐DNA (Yokoi et al. 2019). They showed a CD63‐mediated interaction between micronuclei and multivesicular bodies (Yokoi et al. 2019). However, micronuclei are tiny buds of the nucleus resulting from DNA damage found in cancer cells and cannot explain the presence of DNA in EVs derived from normal cells nor the presence of vesicular mtDNA (Figure 2). Similar to the passive loading of EV RNA, DNA might be passively packaged into EVs to control cellular homeostasis. Another study proposed that DIAPH3‐depleted amoeboid cells with nuclear shape instability secrete non‐apoptotic EVs with nuclear material (Reis‐Sobreiro et al. 2018). However, the authors did not physically and biochemically characterize the isolated EVs to identify which EV population carried nuclear DNA (Reis‐Sobreiro et al. 2018). In a study on microvesicles, it has been demonstrated that dsDNA recruitment in tumour‐associated microvesicles is regulated by ARF6 GTP/GDP cycling (Figure 2) (Clancy et al. 2022). They suggest that the cytoplasmic DNA sensor cGAS plays a vital role in specific DNA loading in microvesicles and not in another vesicle compartment such as the intermediate organelle, the amphisome (Clancy et al. 2022).

It is essential to understand that the packaging of DNA EVs is specific to the biological context. Identifying the circumstances under which cells release DNA into EVs is crucial to comprehend the mechanisms of EV‐DNA packaging. This includes identifying specific DNA‐binding proteins or structures/patterns in DNA that might be responsible for directing DNA to EVs. From a clinical perspective, cancer cells may employ distinct pathways that facilitate the loading of tumour DNA into EVs. Additionally, cancer EV‐DNA may carry unique DNA sequences or epigenetic patterns recognized by the sorting machinery during EV biogenesis. Consequently, targeting the mechanisms involved in cancer EV‐DNA sorting and packaging could selectively disrupt the homeostasis and stability of cancer cells, potentially eliciting an anti‐tumour immune response, as will be discussed in the following sections.

## 6 EV‐DNA: Uptake and Functions

### 6.1 EV‐DNA of Nuclear Origin

**FIGURE 4:**
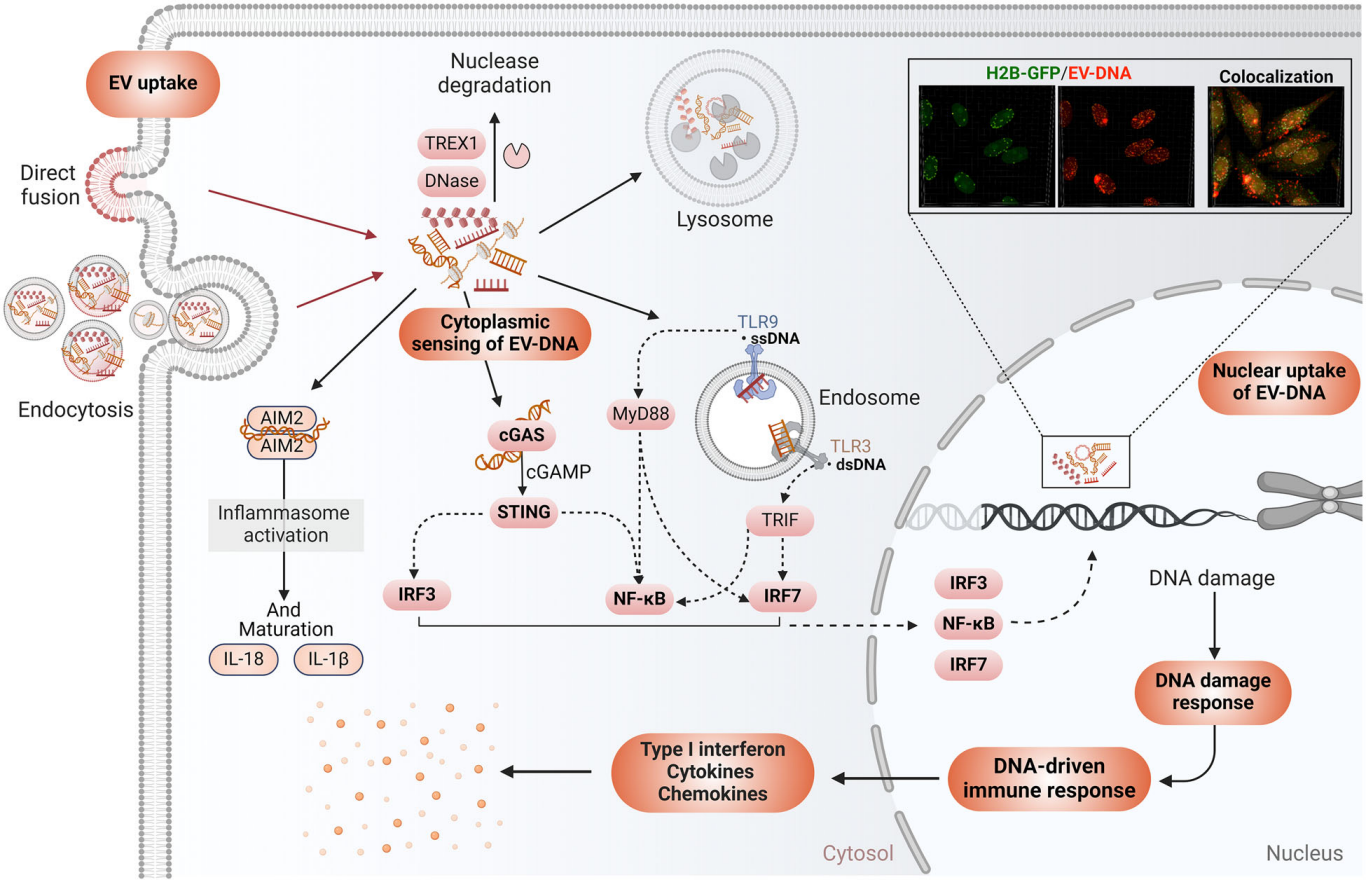
EV DNA uptake and its interaction with recipient cells compartments. EVs can deliver their cargo with multiple mechanisms (not all are shown here), including direct fusion with cell membranes and endocytosis. In the cytoplasm, internalized EV‐DNA interacts with DNA sensors absent in melanoma 2 (AIM2) and can also be recognized by pattern recognition receptors such as Toll‐like receptors (TLRs). However, the interaction between EV‐DNA and the DNA sensing machinery cGAS/STING is the most studied. Vesicular DNA mediates the stimulation of the cGAS/STING pathway, leading to the recruitment and activation of transcription factors IRF3/7 and NF‐κB. Nuclear translocation of IRFs and NF‐κB initiates the transcription of the type I interferon gene and many other cytokine and chemokine genes to trigger an (EV)‐DNA‐driven innate immune response. A part of delivered EV‐DNA is directed to lysosomes or degraded by cytoplasmic nucleases such as TREX1 and DNases. DNA cargo not cleared in the cytoplasm reaches the nucleus, as shown in the 3D confocal pictures. Part of EV‐DNA detected in the nucleus is associated with histones like H2B, which might protect it from cytoplasmic degradation. Nuclear uptake of EV‐DNA can happen, but the role of this DNA in the nucleus remains to be determined. The 3D confocal images are reused from Ghanam et al. (2023) with permission from Springer Nature.

Studies dealing with the function of internalized fragments of EV‐DNA have mostly focused on the activation of DNA sensors such as cGAS and absent in melanoma 2 (AIM2), and DNA pattern recognition like TLR (Figure 4). It was recently hypothesized that cells package and release harmful DNA into EVs to prevent cytoplasmic accumulation and maintain their normal homeostasis. A few recent studies have provided insight into this area (Takahashi et al. 2017; Zhao et al. 2021). Transient inhibition of EV release using multiple siRNAs targeting ALIX and RAB27a mRNAs resulted in the accumulation of DNA fragments in the cytoplasm with concomitant activation of the STING machinery (Takahashi et al. 2017). The authors suggested the cytoplasmic‐DNA‐driven immune response leads to reactive oxygen species (ROS)‐dependent DNA damage response, which finally promotes the apoptosis or senescence‐like cell‐cycle arrest in ALIX and RAB27a‐depleted cells (Takahashi et al. 2017). Finally, there were still some drawbacks to this study, such as the use of only PCR to quantify cytoplasmic DNA. Nuclear DNA in EVs is linked with inflammatory bowel conditions like Crohn's disease (Zhao et al. 2021). These EVs mostly originated from damaged intestinal epithelial cells (Zhao et al. 2021). EV‐DNA concentration was higher in murine colitis and active Crohn's disease patients than in patients in remission. It positively correlated with disease activity, suggesting that the secretion and transport of nuclear DNA fragments mainly depend on EV release (Zhao et al. 2021). Small EVs from Crohn's disease patients and triple‐negative breast cancer cells were able to activate the cGAS/STING pathway in macrophages (Zhao et al. 2021; Tkach et al. 2022). Further, inhibition of EV release resulted in improvement of prognosis of murine colitis (Zhao et al. 2021). In acute myeloid leukaemia (AML), EVs can transfer chromatin‐like structures (EV‐chromatin) to bone marrow mesenchymal stem cells and modulate their behaviour (Ghanam et al. 2023). The EV‐DNA was associated with histones and other proteins such as S100s and serpin A (Ghanam et al. 2023). Independently of DNA sensing machinery activation, this study demonstrated that AML‐EV‐chromatin structures can regulate the proliferation of bone marrow mesenchymal stem cells through non‐mutational inactivation of p53, potentially involving S100 proteins associated with EV‐chromatin (Ghanam et al. 2023).

### 6.2 EV‐DNA of Mitochondrial Origin

The levels of mtDNA and its associated mutations have garnered significant attention in cancer research due to their correlation with disease development and resistance to therapy, which has been extensively discussed in previous sections (Zong et al. 2016; Weerts et al. 2016). Multiple research groups have documented the presence of mtDNA in EVs (Table 1). Notably, small EVs have the ability to transfer the entire mitochondrial genome to stem‐like breast cancer cells with impaired metabolism, as demonstrated by Sansone et al. (2017). The uptake of mtDNA aids in restoring the metabolic activity of breast stem cancer cells, ultimately facilitating their escape from dormancy and promoting tumour progression. Furthermore, it has been observed that mtDNA‐enriched EVs act as DAMP molecules in various conditions, such as frailty (Byappanahalli et al. 2023), aging (Lazo et al. 2021), and liver fibrosis (Li et al. 2022).

## 7 EV‐DNA for Therapeutic Applications

**FIGURE 5:**
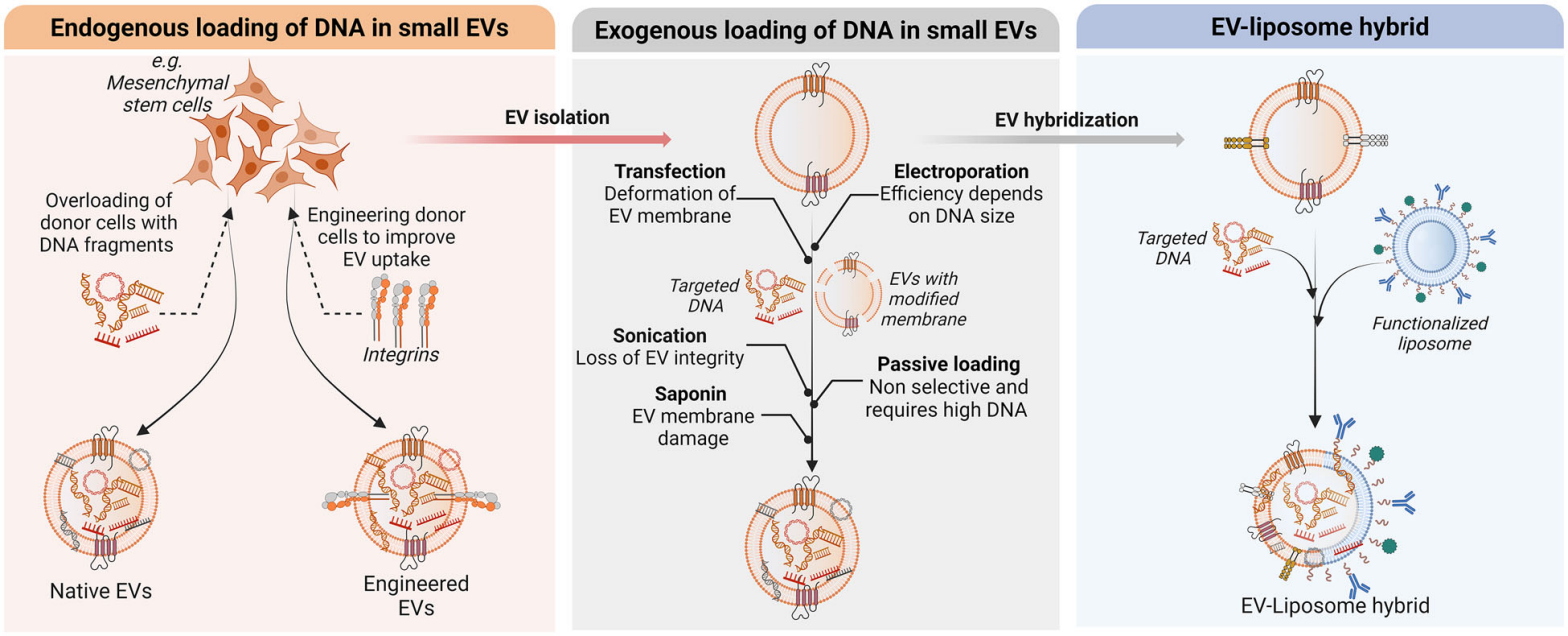
Modalities of DNA loading into small EVs. Cells such as mesenchymal stem cells, fibroblasts, endothelial, and dendritic cells are potential sources of native and engineered EVs. Native EVs with DNA can be endogenously produced after overloading of donor cells (e.g., mesenchymal stem cells) with desired DNA fragments. Engineered cells that overexpress specific transmembrane proteins, like the integrin family, can serve as a source to produce decorated EVs to improve their targeting efficiency and pharmacokinetics. Post‐isolation loading (exogenous) methods also hold promise in developing controlled and reproducible processes of DNA incorporation into EVs. For this purpose, various loading methods such as electroporation, transfection, and sonication can be utilized, despite being adapted and optimized to introduce nucleic acids into cells, not into EVs. Therefore, parameters such as EV heterogeneity, EV‐membrane and EV integrity, and the size of targeted DNA should be considered in the future to adjust the existing methods to extracellular vesicles. The capacity of EVs can be improved by fusing them with synthetic nanocarriers such as liposomes.

The field of targeted or “smart” drug delivery is rapidly expanding with various delivery systems, including colloidal systems like liposomes, polymers, and cellular and subcellular systems. The goal is to deliver therapy to specific organs, tissues, or cells with high efficiency and fewer toxic effects. In addition to the current array of nanocarriers, there is a growing interest in EVs as biological nanoparticles for smart drug delivery due to their natural role in facilitating the intercellular transport of various molecules (O'Brien et al. 2020; Herrmann et al. 2021; Cheng and Hill 2022; Möller and Lobb 2020; Zhang et al. 2020; Zhu et al. 2022; Belhadj et al. 2020). Particularly, EVs can serve as vehicles to deliver nucleic acids, drawing attention towards their use for gene therapy and RNA interference applications, as it has extensively been reviewed by O'Brien et al. (2020). The abovementioned studies have put forth the idea of using EVs over conventional synthetic carriers for the delivery of nucleic acids since they are less immunogenic and toxic than viruses due to their physiological origin. The biodistribution and organotropism of EVs seem regulated by their surface markers, which can direct them towards specific organs (Hoshino et al. 2015). Recipient cells can internalize EVs in different ways, like phagocytosis, macropinocytosis, and receptor‐mediated endocytosis. Moreover, large‐scale and cost‐effective production of EVs and the potential assessment of batch‐to‐batch variations would be possible with the use of high productivity and sensitivity methods for isolation and characterization. Nucleic acids can be loaded into EVs using existing loading approaches such as transfection and electroporation (Figure 5). However, it remains to be determined which loading method is compatible with each EV population, as changes in physical and biochemical characteristics between vesicle subpopulations might impair the loading efficiency, as we have summarized in Figure 5.

**FIGURE BOX 2:**
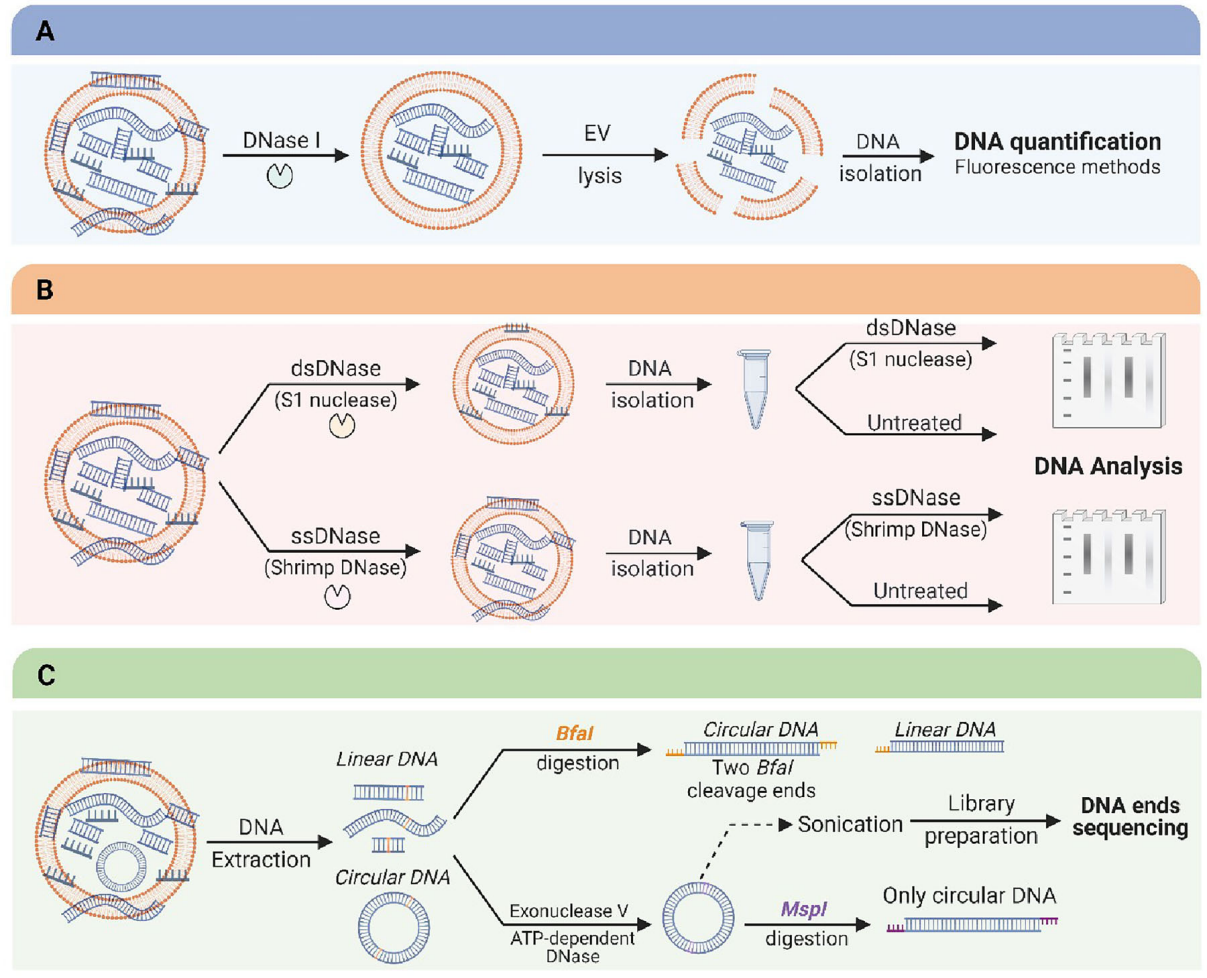
Experimental strategies for studying EV‐DNA: (a) localization and (b–c) topology.

### 7.1 Intracellular Loading of EVs With DNA and Nucleic Acids: Endogenous Loading

EV‐DNA and EV‐chromatin can reach the nucleus of the recipient cells, but the implication of this nuclear transfer is yet to be determined. Fischer et al. (2016) showed the ability of EVs to transfer foreign genes into recipient cells horizontally. More precisely, human bone marrow‐derived mesenchymal stromal cells have successfully integrated *Arabidopsis thaliana* DNA delivered by EVs (Fischer et al. 2016). EVs can transfer the sex‐determining region of Y (SRY) DNAs to cells that do not endogenously express SRY protein (Cai et al. 2015). Authors incubated EVs from SRY‐transfected HEK293 cells with monocytes and human umbilical vein endothelial cells (HUVECs), which have been found to express newly synthesized SRY protein (Cai et al. 2015). This transfer has increased the expression of CD11a and ICAM‐1 adherence factors in THP‐1 and HUVECs (Cai et al. 2015). Intriguingly, the injection of SRY EVs into ApoE‐/‐ mice resulted in the acceleration of atherosclerosis (Cai et al. 2015). The same authors have previously reported that K562‐derived EVs could transfer the BCR/ABL hybrid gene, involved in the pathogenesis of chronic myeloid leukaemia, from K562 to HEK293 cells or neutrophils (Cai et al. 2013). Another aspect of EV‐mediated gene transfer is the delivery of viral DNA from infected to non‐infected cells. Saari et al. (2020) studied the kinetics of EV secretion by cancer cells infected with an oncolytic adenovirus. They found that infected cells release normal EVs before the lytic phase (virions release), precluding the apoptotic origin of these EVs (Saari et al. 2020). EVs derived from infected cells were able to deliver viral genomes to initiate infection in other cancer cells (Saari et al. 2020). As per the authors, carrying virus components helps viruses escape the immune system, providing an alternative entry pathway into target cells (Saari et al. 2020).

Similarly, small EVs and microvesicles can encapsulate reporter proteins and mRNA, but de novo expression of reporter protein has been only observed for plasmid DNA delivered via microvesicles (Kanada et al. 2015). Additionally, microvesicles were able to transfer functional plasmid DNA encoding Cre recombinase in transgenic Cre‐lox reporter mice (Kanada et al. 2015). Again, the use of ultracentrifugation as an isolation method was one major limitation in this study besides the absence of proper characterization of EV populations. Serial ultracentrifugation steps can result in morphological changes and sample loss in isolated EVs (Sidhom et al. 2020; Liangsupree et al. 2021; Furi et al. 2017; Lobb et al. 2015).

Zhao et al. (2021) proved that mesenchymal stem cell EVs carry entire mtDNA. Once these EVs target the recipient cells, mtDNA is able to fuse with recipient cell mitochondria and upregulate mtDNA‐coded genes and mitochondrial oxidative phosphorylation. Therefore, mtDNA, together with other material carried by mesenchymal stem cell EVs, attenuates mitochondrial damage and could thus be used as therapeutics in regenerative medicine (Zhao et al. 2021).

### 7.2 Post‐Isolation Loading of EVs With DNA and Nucleic Acids: Exogenous Loading

Post‐isolation loading of EVs with DNA fragments larger than siRNA or miRNA has also stoked interest. Using electroporation, Lamichhane et al. claimed to have successfully loaded DNA of different lengths into EVs with an average of hundreds of molecules per vesicle (Lamichhane et al. 2015). They used electroporation as a loading method, and they reported that the efficiency largely depends on the sizes of both DNA and EVs (Lamichhane et al. 2015). To prevent immune clearance, placenta‐derived EVs were recently investigated for their ability to carry plasmid DNA using electroporation and passive incubation as loading strategies (Kang et al. 2023). The authors show successful loading of plasmid DNA into both large and small EVs with more encapsulation efficiency in large EVs. They have again highlighted that the loading efficiency is regulated by DNA size and concentration (Kang et al. 2023).

### 7.3 EV‐liposome Hybrids for Nucleic Acids Delivery

EVs containing DNA of interest hold great potential as natural therapeutic tools for cancer and other disorders to deliver therapeutic genes to target cells and tissues efficiently. Encapsulating DNA within EVs can protect it from cytoplasmic degradation and functionally deliver it to specific cell types. The challenge is how we can engineer EVs to enhance their targeting specificity and efficiency for delivering therapeutic DNA to desired cell populations. Once optimized, this approach opens up possibilities for treating genetic disorders, cancer, and other diseases at the molecular level by gene replacement, gene silencing, or gene editing approaches. The current findings that EVs containing DNA can also be utilized for immune modulation by delivering immunomodulatory genes or antigens to immune cells hold promise for immunotherapy and treating immune‐related disorders (Buzas 2023). As with any therapeutic approach, ensuring the safety and efficacy of EV‐associated DNA is crucial. Therefore, to minimize side effects, it is essential to thoroughly optimize and evaluate the therapeutic dosage, administration route, potential off‐target effects, immunogenicity, and long‐term consequences of EV‐mediated DNA delivery.

Further, establishing scalable and reproducible methods for manufacturing EV‐associated DNA therapeutics is critical for their clinical translation. The success of all these steps will help in the smooth development and regulatory approval of EV‐associated DNA therapeutics. In the future, more research and development efforts are required to harness the potential of extracellular vesicle‐associated DNA for therapeutic purposes. Continued progress in understanding the biology of EVs, optimizing delivery strategies, and addressing the associated challenges will pave the way for their successful translation into clinical applications. Despite extensive research, including some ongoing clinical trials, the successful implementation of an EV‐based gene delivery system is yet to be achieved. The endogenous loading of nucleic acids is less efficient and, so far, a non‐controlled process as the EV cargo changes from one batch to another. More importantly, as discussed in the above sections, the mechanisms behind sorting and packaging nucleic acids into EVs still need to be discovered. Therefore, harnessing the biogenesis properties to load EVs with desired nucleic acids endogenously remains enigmatic, even with genetically modified cells as a source of EVs. Conversely, exogenous loading methods are generally more elaborate but must be adapted for EV applications. For this purpose, newly emerging approaches such as cellular nanoporation have effectively increased the loading yield of mRNA into small EVs (Yang et al. 2020). Another exciting new avenue is the fusion of pre‐loaded liposomes with EVs to enhance the loading and organotropism abilities (Fuhrmann et al. 2015; De Jong et al. 2019).

## 8 Conclusions and Perspectives

While the exchange of genetic material via EVs is a consensus in prokaryotes and lower eukaryotes (Ghanam et al. 2022), many questions remain to be resolved before fully decoding this complex language in higher organisms. We underlined various questions and concerns regarding the EV‐DNA structure, mechanism(s), and circumstances of its packaging, uptake, and function(s). We also highlighted that the current biomarker studies on EV‐DNA primarily focus on detecting cancer‐related mutations. In contrast, the fragmentomic and topologic characteristics of EV‐DNA continue to be largely unexplored.

Different modalities of cellular EV uptake, including endocytosis, direct fusion with the plasma membrane, or receptor‐mediated interactions, have been proposed. However, none have been reported to be specific to DNA‐carrying EVs. A key issue here is overcoming technical and biological challenges by identifying the unique DNA‐carrying EV population(s) and emphasizing their uptake mechanism. A deeper understanding of the molecular pathways involved in the uptake of DNA‐carrying EVs helps shed light on the cellular processes triggered by this DNA in recipient cells.

Regarding EV‐DNA‐mediated signalling, the interaction of EV‐DNA with cytoplasmic DNA sensors other than cGAS/STING and the consequence of this association await clarification. Moreover, although EV‐DNA can be transferred to the nucleus, whether this DNA is integrated into the host genome and transcribed to functional RNAs remains enigmatic.

Until now, characterization of specific recruitment mechanisms of DNA into EVs has proved difficult, probably owing to the lack of technologies allowing the analysis of DNA's interaction with the EV biogenesis machinery, such as syntenin–ALIX, tetraspanin, and ceramide pathways. Technologically, future studies should be devoted to implementing new approaches, such as super‐resolution microscopy, to track EV‐DNA sorting and loading into EVs at the nanoscale level. In this regard, super‐resolution microscopy techniques such as SMLM and MINFLUX are gaining importance (Ghanam et al. 2023).

Research and development efforts are ongoing to harness the potential of extracellular vesicle‐associated DNA as a promising therapeutic modality. Continued progress in understanding the biology of EVs, optimizing delivery strategies, and addressing the associated challenges will pave the way for their successful translation into clinical applications.

## AUTHOR CONTRIBUTIONS

**Jamal Ghanam**: Conceptualization (lead), methodology (lead), validation (lead), visualization (lead), writing – original draft (lead), writing – review and editing (lead). **Kristína Lichá**: Visualization (supporting), writing – original draft (equal), writing – review and editing (supporting). **Venkatesh Kumar Chetty**: Writing – original draft (supporting), writing – review and editing (supporting). **Ommolbanin Asad Pour**: Writing – original draft (supporting), writing – review and editing (supporting). **Dirk Reinhardt**: Methodology(equal), validation(equal), writing – original draft(supporting), writing – review and editing(supporting). **Barbora Tamášová**: Writing – review and editing (supporting). **Jan Lötvall**: Methodology(equal), validation(equal), writing – original draft(supporting), writing – review and editing(supporting). **Basant Kumar Thakur**: Conceptualization(lead), funding acquisition(lead), methodology(equal), validation(equal), visualization(supporting), writing – original draft(supporting), writing – review and editing(supporting).

## Ethics Statement

Not applicable.

## Conflicts of Interest

The authors declare no conflicts of interest.

## Data Availability

Not applicable.
